# The pediatric disease spectrum in emergency departments across Pakistan: data from a pilot surveillance system

**DOI:** 10.1186/1471-227X-15-S2-S11

**Published:** 2015-12-11

**Authors:** Huba Atiq, Emaduddin Siddiqui, Surriya Bano, Asher Feroze, Ghazala Kazi, Jabeen Fayyaz, Shivam Gupta, Juanid A Razzak, Adnan A Hyder, Asad I Mian

**Affiliations:** 1 Department of Emergency Medicine, Aga Khan University, Pakistan; 2 Johns Hopkins International Injury Research Unit, Department of International Health, Johns Hopkins Bloomberg School of Public Health, Baltimore Maryland, USA; 3 Department of Emergency Medicine, Johns Hopkins School of Medicine, Baltimore, Maryland, USA; 4 Department of Paediatrics and Child Health, Aga Khan University, Pakistan; 5 The author was affiliated with the Department of Emergency Medicine, Aga Khan University, Karachi, Pakistan at the time when study was conducted

**Keywords:** Pediatric Emergency, Pakistan, Low to Middle Income Countries, Children, Pediatric Injury, Acute Illness, Pediatric Emergency Room

## Abstract

**Background:**

There is an increasing number of urgently ill and injured children being seen in emergency departments (ED) of developing countries. The pediatric disease burden in EDs across Pakistan is generally unknown. Our main objective was to determine the spectrum of disease and injury among children seen in EDs in Pakistan through a nationwide ED-based surveillance system.

**Methods:**

Through the Pakistan National Emergency Department Surveillance (Pak-NEDS), data were collected from November 2010 to March 2011 in seven major tertiary care centers representing all provinces of Pakistan. These included five public and two private hospitals, with a collective annual census of over one million ED encounters.

**Results:**

Of 25,052 children registered in Pak-NEDS (10% of all patients seen): 61% were male, 13% under 5 years, while almost 65% were between 10 to < 16 years. The majority (90%) were seen in public hospital EDs. About half the patients were discharged from the EDs, 9% admitted to hospitals and only 1.3% died in the EDs. Injury (39%) was the most common presenting complaint, followed by fever/malaise (19%) and gastrointestinal symptoms (18%). Injury was more likely in males vs. females (43% vs. 33%; p < 0.001), with a peak presentation in the 5-12 year age group (45%).

**Conclusions:**

Pediatric patients constitute a smaller proportion among general ED users in Pakistan. Injury is the most common presenting complaint for children seen in the ED. These data will help in resource allocation for cost effective pediatric ED service delivery systems. Prospective longer duration surveillance is needed in more representative pediatric EDs across Pakistan.

## Background

Each year an increasing number of critically ill children present to emergency departments (EDs) of tertiary-care hospitals of both developing and developed countries [1-3]. The World Health Organization (WHO) conducted a study in 21 hospitals in low-income countries like Bangladesh, Dominican Republic, Ethiopia, and Indonesia, and reported that 90% of children had severe forms of common infectious diseases, especially pneumonia, diarrhea, sepsis, malaria and meningitis, often complicated with chronic malnutrition [2]. These preventable infectious disease spectrum are the major cause of death and disability in South Asia, contributing to 3.7 million deaths in children, over 60% of whom are under 5 years old [5]. How many of these can be readily identified and managed in EDs is unknown. In 2010 there were over 25 million ED visits for children younger than 18 years in the United States; almost 90% of these visits resulted in release from the ED after treatment [4]. Boys accounted for a slightly larger proportion of ED visits [4]. The mean age of children being seen in the EDs was approximately 7 years; while infants less than one year accounted for 5% of pediatric ED visits [4]. Injuries and poisoning were common presenting complaints for older children more than 5 years old, while respiratory-related issues were more frequent in the infant age group [4]. Literature from low-income countries suggests that pediatric emergency departments encounter more pediatric patients with medical problems, while a general ED is more likely to treat children with injuries and other musculoskeletal complaints [1,3,6-8]. The clinical course of children in a pediatric ED is also unique when compared to children in a general ED; children in pediatric ED tend to be sicker [6-8].

In low- and middle-income countries (LMICs), ill or injured children are often taken to the nearest community-based hospital, irrespective of resources or the ability of the ED to manage the case [1,3]. These children may have fatal diseases or arrive too late in the course of the illness, which contributes to rising mortality [3]. According to a recent WHO report, unintentional injuries kill approximately 830,000 children every year, with more than 95% of those occurring in LMICs [13,14]. A global childhood unintentional injuries surveillance done in five major cities of LMICs suggested that falls (50.4%) and road traffic injuries (16.4%) were the most common and affect boys more often than girls (64.7% vs. 35.3%) [13,14]. Studies on the pediatric ED disease spectrum in developing countries show that common presenting complaints tend to be fever, upper respiratory tract infection and diarrhea [9-12], and common final diagnoses are upper respiratory tract infection (26.7%), viral syndrome (13.1%) and gastroenteritis (10.7%) [9-12]. Therefore, the majority of chief complaints and final diagnoses are infection-related (up to 64%) [9-12].

Pediatric emergencies can burden limited ED resources, and this is particularly concerning for LMICs. Emergency department related disease spectrum for children across Pakistan is unavailable and thus the case mix of the population is unknown. Knowing the disease burden in terms of frequency of illness or injury in the ED will help plan an effective service delivery system in resource limited settings. The establishment of the Pakistan National Emergency Department Surveillance (Pak-NEDS) was a step towards capturing this information. The primary objective of this study was to determine the burden of pediatric diseases presenting to the EDs of major tertiary care centers across Pakistan. A secondary objective was to investigate if factors such as age, gender, location or type of ED were different among the pediatric diseases being identified through the Pak-NEDS system.

## Methods

### Study design and patient enrollment

The Pakistan National Emergency Departments Surveillance (Pak-NEDS) was a pilot active surveillance done from November 2010 to March 2011. The study sites were seven major EDs in Pakistan: Aga Khan University (AKU) and Jinnah Post-graduate Medical Center in Karachi, Benazir Bhutto Hospital in Rawalpindi, Lady Reading Hospital in Peshawar, Mayo Hospital in Lahore, Sandeman Provincial Hospital in Quetta, and Shifa International Hospital in Islamabad. All the sites are tertiary care urban centers; AKU and Shifa International Hospital are private hospitals while the rest are public hospitals. AKU was the main coordinating center for the study. All participating EDs are housed in major hospitals in their respective cities, and serve the entire population of those cities. The study participants for Pak-NEDS were males and females of all pediatric age groups presenting to the EDs of participating sites to seek care for any reason, illness or injury. All patients under 16 years were included as cases as it was the age cut off for pediatrics. Ethical approval from all relevant committees was obtained from all participating sites prior to study initiation.

### Data collection tool

A one-page standardized questionnaire was developed based on the ambulatory care survey tool of the Centers for Disease Control and Prevention, USA and previous surveillance work done in Pakistan [11]. The data collection tool was finalized after consultation with the ED heads of all institutions. The tool had questions related to patient demographics like age, gender, ethnicity, presenting complaints, treatment and management provided in the ED and disposition from the ED.

### Study procedure

Data collectors were specifically hired and trained for this study and they worked in three shifts providing 24-hours coverage. Patients were recruited upon registration in the ED. All data was gathered while the patient was in ED, and there was no post ED patient-contact in this study. Data collection was done from the patient or next of kin interviews and ED records. Hard copies of the data collection tool were then sent to the coordinating center at AKUH.

### Data entry and analysis

Data were entered at AKUH using EpiInfo version 3.3.2 and analysis was done using SPSS version 19. The burden of disease in the pediatric (<16 years) population was analyzed based on the list of presenting complaints and provisional or final diagnoses in the questionnaire. Descriptive data were obtained and reported as mean and median for quantitative variables and proportions for qualitative variables.

## Results

There were 25,052 (9.7%) patients who were less than16 years of age in Pak-NEDS; the mean age of pediatric patients enrolled was 10.5 years (SD ± 4.3 years), with 61% males (Table 1). The majority of patients (65%) were between 10 to < 16 years. Patient's gender stratified by age revealed that in all age groups the proportion of males was higher (60% for above 5 yrs; 57% for toddlers and 56% for infants; p < 0.008). The vast majority of the pediatric patients in Pak-NEDS presented at public hospitals (89%). Almost 97% were brought to the ED by non-ambulance means, while a much smaller proportion (3.1%) were brought by ambulance services. The median time to ED presentation was 170 minutes [Inter-quartile range (IQR) 40-721 minutes]. About 70% were evaluated by a medical officer, 63.8% by a nurse or midwife, and a small proportion (0.7%) was evaluated by an attending physician (Table 1).

**Table 1:** Patient Characteristics of children under 16 years enrolled in Pakistan National Emergency Department Surveillance (n = 25052)

Variables	n	%
**Age Group**		
< 5 years	3309	13.2
5 - <10 years	5357	21.4
10 - <16 years	16386	65.4
**Gender (n = 24715)**		
Male	15102	61.1
Female	9613	38.9
**Mode of arrival (n = 23925)**		
Ambulance	744	3.1
Non-ambulance	23181	96.9
**Type of hospital**		
Public hospital	22303	89.0
Private hospital	2749	11.0
Type of care provider* (n = 23664)		
Paramedic	10578	44.7
House officer/intern	7860	33.2
PG trainee/Resident	3091	13.1
Medical Officer	16549	69.9
Nurse/midwife	15086	63.8
Attending physician	175	0.7
**Disposition (n = 18772)**		
Discharged from ED	8980	47.8
Ward admission	1658	8.8
ICU/HDU admission	125	0.7
Observation unit	2211	11.8
Follow-up	4563	24.3
Expired	250	1.3
Others (LAMA/LWBS/Referred out)**	985	5.2

* Multiple response variables

**LAMA, Left against medical advice; LWBS, Left without being seen.

Information pertaining to patient status at time of arrival was lacking in 12% of cases; 0.5% were reported dead at the time of arrival to the ED. Data on ED disposition was available for 74% of patients; almost half were discharged (48%), 8.9% were admitted and 1.3% of cases were reported to have died while in the ED (Table 1). This represents a mortality rate of 1300 per 100,000 pediatric patients seen in the ED.

The "presenting complaint" variable was used to analyze the disease spectrum among the pediatric age group by the ED physicians (Table 2). Of the 22,047 cases, injury was the most frequent complaint reported at the time of arrival in ED (39.4%), followed by constitutional symptoms such as fever and malaise (18.9%) and gastrointestinal symptoms (18.3%). The presenting complaints were further stratified by gender, age, province and the type of hospital (Table 3). Females had a higher percentage of gastrointestinal, respiratory and neurological presenting complaints as compared to males, while males had comparatively higher percentage related to injury (43.4% as compared to 33% in female; p < 0.001). Presenting complaints relating to constitutional (fever, malaise, etc.), cardiovascular and musculoskeletal symptoms were similar among both genders. When stratified by age, there were fewer cases of neurological and musculoskeletal symptoms among children under 5 (4.6% and 5.8% respectively) as compared to older age groups. Similarly, for cardiovascular symptoms, there were fewer children under 5 years with these presenting complaints (7.4%) versus the other age groups (Table 3). Children under 5 years were more likely to present with injury and constitutional-related presenting complaints, than older age groups (p < 0.001). Of the children with injury as a chief presenting complaint, 42% were seen at a public hospital ED, whereas children with gastrointestinal, respiratory, and neurological emergencies reported mostly to private hospitals (p < 0.001) (Table 3). A significant difference was observed in case mortality rates in the EDs, between public and private facilities (25 per 1000 children in a private ED vs. 12 per 1000 children in a public ED; p < 0.001).

**Table 2:** Top 10 presenting complaints for pediatric patients in Pakistan National Emergency Department Surveillance (N = 22047)

Presenting Complaints for Patients < 16 years	N	%
Injury	8680	39.4
Constitutional*	4160	18.9
Gastrointestinal	4025	18.3
Respiratory	3018	13.7
Neurological	2825	12.8
Cardiovascular	1391	6.3
Musculoskeletal	1332	6
Eye & Otolaryngology	500	2.3
Genitourinary	457	2.1
Others^+^	679	3.1

*Fever, lethargy, lassitude, weakness, loss of appetite, etc.

^+^Skin, Psychiatric

**Table 3:** Presenting complaints stratified by patient characteristics for pediatric patients under 16 years of age in Pakistan National Emergency Department Surveillance (n = 22047)

	Disease Spectrum n(%)							
	
	Constitutional	Cardiovascular	Gastrointestinal	Respiratory	Neurological	Musculoskeletal	Injury	Total
**Gender**	4123	1358	3965	2997	2815	1315	8573	**21777**

Male	2561 (19.1)	754 (5.6)	2203 (16.4)	1657 (12.4)	1492 (11.1)	754 (5.6)	5810 (43.4)	**13396**
Female	1562 (18.6)	604 (7.2)	1762 (21)	1340 (16)	1323 (15.8)	561 (6.7)	2763 (33)	**8381**
P-value	NS	< 0.001	< 0.001	< 0.001	< 0.001	0.001	< 0.001	**--**

**Age Group**	4160	1391	4025	3018	2825	1332	8680	**22047**

								
<5 years	729 (24.9)	218 (7.4)	566 (19.3)	390 (13.3)	135 (4.6)	170 (5.8)	1126 (38.4)	**2931**
5-9 years	960 (20.0)	274 (5.7)	715 (14.9)	591 (12.3)	395 (8.2)	248 (5.2)	2330 (48.6)	**4790**
10-15 years	2471 (17.2)	899 (6.3)	2744 (19.2)	2037 (14.2)	2295 (16.0)	914 (6.4)	5224 (36.5)	**14326**
P-value	< 0.001	0.010	< 0.001	< 0.01	< 0.001	< 0.01	< 0.001	**--**

**Type of hospital**	4160	1391	4025	3018	2825	1332	8680	**22047**

Public hospital	3568 (18.3)	1211 (6.2)	3358 (17.2)	2453 (12.6)	2185 (11.2)	1220 (6.3)	8168 (41.9)	**19505**
Private hospital	592 (23.3)	180 (7.1)	667 (26.2)	565 (22.2)	640 (25.2)	112 (4.4)	512 (20.1)	**2542**
P-value	<0.001	0.089	< 0.001	< 0.001	< 0.001	< 0.001	< 0.001	**--**

## Discussion

This study is the first of its kind to describe the pediatric disease spectrum in EDs across Pakistan. The disease frequency revealed in this study was from general EDs across the country and thus might not be wholly representative of the actual disease spectrum as observed in dedicated pediatric EDs [5,10-12]. What is noteworthy in this study is that only 10% of patients enrolled through Pak-NEDS belonged to the pediatric age group. One reason for this could be that children might be taken to EDs that deal with children rather than being brought to general EDs. The literature also suggests that a dedicated pediatric ED encounters more patients with medical problems while a general ED is more likely to treat children with injuries and other musculoskeletal complaints [1,6,7]. The clinical course of children in a pediatric ED is also unique when compared to children in a general ED; for example, children in a pediatric ED tend to be sicker at admission [6-10]. Furthermore, the burden of chronic illness in LMICs is higher in a pediatric ED due to referral from the primary physicians or sub-specialists [15,16]. Pediatric EDs are typically in children's hospitals versus pediatric sections in general EDs. How this impacts pediatric emergency care in resource-limited settings is not entirely clear, as there are likely to be even fewer dedicated children's hospitals with pediatric emergencies in such settings [7].

Injuries accounted for almost half of the presenting complaints to the EDs [8,17] which, considering the high morbidity and mortality related to trauma, poses a significant burden on hospitals [13,14,17-20]. Although this study was different from the previous ED-based surveillance study focused specifically on childhood unintentional injuries in LMICs including Pakistan [17-20], both studies revealed that boys were more likely to be injured. Most injuries were minor; however, the predominance of head, face and limb injuries might lead to significant future morbidity and disability. Furthermore, the possibility that some trauma was non-accidental or intentional cannot be overlooked.

Pediatric infection-related ailments were also relatively frequent presenting complaints. This is also consistent with literature from Pakistan and other LMICs as presenting complaints of constitutional symptoms (like fever, malaise, lethargy), gastrointestinal (diarrhea, vomiting), acute respiratory and neurological symptom could all indicate infectious etiology [21-23]. In fact, Pakistan is one of five countries with the highest under five mortality due to infectious disease [2-5,24].

It is important to keep in mind that Pakistan has few established EDs that are led by formally trained emergency medicine staff [25-29]. EDs (commonly called casualty rooms) are primarily run by junior medical officers with limited expertise in delivering pediatric care [25,26,29]. These medical officers are only capable of handling the minor illnesses while more complicated and serious cases are being immediately referred to specialist pediatric residents on-call or to other pediatric hospital [26,27]. The EDs at basic health units or the primary health centers in rural or peri-urban vicinities are run by paramedics, nurses or health workers with no or minimal training in pediatric care [26,27]. Consistent with this, most patients in this study were either seen by a medical officer or paramedic staff, with less than 1% of the children being seen by an ED or pediatric consultant [26,27].

In addition to this, the structured pediatric teachings at undergraduate level started in Pakistan in the late 1990's [25-27]. Though there are post-graduate examinations in general pediatrics, pediatric emergency training and examinations are yet to start in the country. This highlights the need for improved training and development of pediatric EDs across the country utilizing a uniform evidence-based medicine approach, particularly for the commonly encountered pediatric ailments in Pakistan.

Ambulance service utilization was found to be negligible in this study, highlighting the need for a well-defined pediatric ambulance service across Pakistan. Reasons for preferential utilization of private transport may include the lack of a well-coordinated nationwide emergency medical service, and lack of awareness regarding usage of ambulances [30-32].

This study has several limitations. The missing data for important variables can be explained by the fact that this was a nationwide surveillance dependent on manpower availability. A centralized computer system keeping track of data collected was also lacking. The diverse and unpredictable nature of ED work flow might have contributed to inefficient data collection. Emergencies like bomb blast across the country result in chaotic situation in EDs which is likely to contribute to problems in data collection. Another limitation was the collection of multiple variable responses. By allowing the patient or family member to report up to three responses, a chief complaint was sometimes difficult to determine in the post hoc analysis. However, for the major analyses, it was a very small percentage of patients that reported any overlap in categories, such as injury and infection. In spite of the above limitations, this study presents a comprehensive data set on almost 25,000 pediatric patient encounters in a few representative EDs across Pakistan.

## Conclusion

Almost half of pediatric presentations to EDs across Pakistan are injury-related. Although alarming, this provides an opportunity to reinforce pediatric trauma management services in EDs, along with screening for potential victims of child abuse. Infection-related complaints are also frequently encountered in pediatric ED visits and this can be a potential opportunity to enhance care by the development and uniform implementation of pediatric emergency medicine guidelines for commonly encountered infectious disease-related problems.

A logical next step would be a surveillance study that is of longer duration, utilizes more comprehensive data collection and more rigorous and robust computer-mediated data entry, and enrolls patients in many more representative EDs across Pakistan, including those of children's hospitals.

## Competing interests

The authors declare that they have no competing interests.

## Authors' contributions

HA, EUS, SB, AF, GK, JF developed the initial draft and analysis plan. HA, EUS, AF and AIM were involved in data analysis and interpretation. JAR, AAH and AIM provided critical review of the draft. JAR and AAH were involved in the overall design and implementation of Pak-NEDS. All authors approved of the final draft.

## Funding statement

The Pak-NEDS study was supported through the "Johns Hopkins International Collaborative Trauma and Injury Research Training Program" [Grant No. D43TW007292] by Fogarty International Center of the United States National Institutes of Health. The content is solely the responsibility of the authors and does not represent the views of Fogarty or NIH.
